# Deep learning for FDG-PET classification in patients with Alzheimer’s disease, dementia with Lewy bodies and their mixed pathology: a solution for diagnostic heterogeneity

**DOI:** 10.3389/fnagi.2026.1780858

**Published:** 2026-03-05

**Authors:** Seonggyu Kim, Seun Jeon, Kwonhwi Cho, Sungwoo Kang, Sungkyu Bang, Byoung Seok Ye, Jong-Min Lee

**Affiliations:** 1 Department of Electronic Engineering, Hanyang University, Seoul, Republic of Korea; 2 Department of Neurology, Yonsei University College of Medicine, Seoul, Republic of Korea; 3 Department of Artificial Intelligence, Hanyang University, Seoul, Republic of Korea; 4 Department of Neurology, Hanyang Seoul Hospital, Hanyang University College of Medicine, Seoul, Republic of Korea; 5 Department of Biomedical Engineering, Hanyang University, Seoul, Republic of Korea

**Keywords:** Alzheimer’s disease and concomitant dementia with Lewy bodies pathology, Alzheimer’s disease classification, deep learning, dementia with Lewy bodies classification, FDG-PET, mixed pathology

## Abstract

**Introduction:**

Mixed pathology of Alzheimer’s disease (AD) and dementia with Lewy bodies (DLB) are frequently observed in patients with cognitive impairment, and complicate clinical diagnosis. We aimed to develop a classification model using ^18^F-fluorodeoxyglucose (FDG) positron emission tomography (PET) to improve diagnostic accuracy for these challenging cases.

**Methods:**

We analyzed FDG-PET images from 277 participants who were categorized into AD, DLB, mixed disease, and healthy control (HC) groups. Deep learning-based classification models were trained on seven binary classification tasks and one multiclass classification task and subsequently integrated into an ensemble model to predict AD, DLB, mixed disease or HC groups.

**Results:**

The model achieved an AUROC of 0.73 (95% CI, 0.69–0.78) for AD, 0.90 (95% CI, 0.89–0.91) for DLB, 0.71 (95% CI, 0.66–0.75) for Mixed, and 0.87 (95% CI, 0.84–0.89) for HC.

**Discussion:**

The model represents the state-of-the-art in automatic FDG-PET-based classification of AD, DLB, Mixed, and HC. This study highlights the utility of FDG-PET as a biomarker for differentiating AD, DLB, Mixed, and HC groups, resolving diagnostic challenges caused by overlapping clinical features.

## 1 Introduction

Lewy body disease (LBD) is the second leading neurodegenerative cause of dementia in older adults, following Alzheimer’s disease (AD) (Kane et al., 2018; Vann Jones and O'Brien, 2014). It is estimated that up to 50% of autopsy-confirmed cases of AD show coexisting Lewy body pathology (Hamilton, 2000; Lippa et al., 1998), and about 50% of autopsy-confirmed patients with Lewy body pathology have coexisting AD pathology (Irwin et al., 2017). Since mixed disease with AD plus Lewy body pathologies shows faster cognitive decline and higher mortality than pure AD (Malek-Ahmadi et al., 2019; Olichney et al., 1998), distinguishing between AD with concomitant Lewy body pathology (mixed disease), pure LBD, and pure AD is crucial for comprehending disease progression, enhancing management strategies, and deciding treatment options (Lemstra et al., 2017).

However, when the pathologies of AD and LBD occurred simultaneously, they influence each other, altering the threshold of each pathology to cause clinical symptoms or cognitive dysfunction, which increases the difficulty of diagnosis (Kapasi et al., 2017). Dementia with Lewy bodies (DLB) is a clinical diagnostic term caused by LBD pathology and is characterized by cognitive fluctuation, visual hallucinations, and motor parkinsonism. Previous studies indicate that when AD and LBD pathologies occur simultaneously, the clinical features of DLB, such as cognitive fluctuations and visual hallucinations tend to decrease (McKeith et al., 2017; van der Zande et al., 2018). As a result, diagnosing AD, LBD, and mixed pathology patient based solely on clinical features is challenging, necessitating the use of biomarkers.

While amyloid positron emission tomography (PET) is a valuable image for diagnosing AD due to the presence of significant β-amyloid deposition in AD patients, approximately half of DLB patients also exhibit β-amyloid deposition similar to AD patients, making it unsuitable as a biomarker for distinguishing DLB from AD (Irwin et al., 2017). Although dopamine transporter (DAT) imaging has shown potential in differentiating DLB from AD (McKeith et al., 2007), studies investigating cases with mixed pathologies are currently lacking. Additionally, other diseases, such as multiple system atrophy and progressive supranuclear palsy, may have abnormal results in DAT imaging, leading to suboptimal specificity of DAT imaging for the diagnosis of LBD. Moreover, normal DAT scans have been reported in patients with DLB (van der Zande et al., 2016), suggesting suboptimal sensitivity of DAT imaging for the diagnosis of DLB.

Previously, metabolic patterns on ^18^F-fluorodeoxyglucose (FDG) PET scans has been observed in patients with DLB distinct from those observed in patients with AD or control subjects (Ye et al., 2020), correlating with symptom severity of DLB (Kang et al., 2021). However, most previous FDG-PET studies have focused on binary classification between AD and DLB (Caminiti et al., 2019; Lim et al., 2009; Mattoli et al., 2023; Minoshima et al., 2001; Mosconi et al., 2008; O'Brien et al., 2014), or multi-class classification involving AD, DLB, healthy control (HC), and other diseases (Etminani et al., 2022; Mosconi et al., 2008; Perovnik et al., 2022; Spehl et al., 2015), without consideration for the mixed disease with AD and DLB.

Since motor parkinsonism, a typical clinical symptom of LBD, is commonly observed in patients with AD, but there is debate over whether it originates from LBD or AD pathology itself (Jang et al., 2020; Kang et al., 2023), rather than defining mixed disease solely based on motor parkinsonism and abnormal DAT imaging as AD with LBD, it would be preferable to use specific clinical symptoms of DLB, such as cognitive fluctuations or visual hallucinations, to define it as AD with DLB. This approach would better address the limitations of current clinical diagnosis. We hypothesize that FDG-PET could serve as a biomarker for differentiating AD, DLB, and mixed disease cases. In this study, we aim to develop a model to classify these groups using FDG-PET data. By developing this model, we anticipate improving diagnostic sensitivity for AD, DLB, HC, and mixed disease cases, which remain challenging to diagnose based on clinical features alone.

## 2 Materials and methods

### 2.1 Dataset

Our dataset is composed of participants who underwent standardized neuropsychological battery called the Seoul Neuropsychological Screening Battery (SNSB), FDG-PET, and T1-weighted (T1w) imaging, classified as follows: 94 with pure AD, 84 with probable DLB, 73 with mixed AD/DLB, and 27 healthy controls. Details on neuropsychological evaluation, imaging data acquisition and criteria for group classification are described below. We partitioned 20% of the data from each class into an independent test set and used the remaining 80% for training and validation, allocating 70% for training and 10% for validation during cross-validation. To overcome data insufficiency in model training, data augmentation, as described below, was applied to the training set.

#### 2.1.1 Clinical assessment—criteria for group classification

Differentiation between dementia and mild cognitive impairment (MCI) was based on the Korean Instrumental Activities Daily Living (K-IADL) (Chin et al., 2018). All patients with Alzheimer’s disease (AD) dementia met the diagnostic guidelines of the 2011 National Institute on Aging and Alzheimer’s Association (NIA-AA) (McKhann et al., 2011), while those with MCI due to AD (MCI-AD) met the NIA-AA workgroup guidelines (Albert et al., 2011). All patients with AD dementia and MCI-AD had positive amyloid positron emission tomography (PET) results, as described below.

The diagnosis of Dementia with Lewy bodies (DLB) was based on the fourth consensus report of the DLB consortium published in 2017 (McKeith et al., 2017). All patients with MCI due to Lewy bodies (MCI-LB) met the 2020 research criteria for MCI-LB (McKeith et al., 2020). All patients with DLB and MCI-LB performed dopamine transporter (DAT) PET. However, because other conditions, including progressive supranuclear palsy, frontotemporal lobar degeneration, and multiple system atrophy, can also present as parkinsonism and show abnormal DAT-PET findings (Hastings et al., 2024; Höglinger et al., 2017), the diagnosis of DLB was not solely based on abnormal DAT-PET results. Since rapid eye movement sleep behavior disorder (RBD) can emerge more than 10 years before the onset of cognitive decline or parkinsonism (Postuma et al., 2019), the diagnosis of DLB was restricted to cases where at least one of the following core clinical features was present: cognitive fluctuations or visual hallucinations. This approach was adopted to prevent misclassification as mixed disease in cases where RBD was present, but AD was considered the primary cause of dementia.

Cognitive fluctuations were considered present if the Mayo Clinic Fluctuation Scale was 3 or higher (Ferman et al., 2004). If patients simultaneously satisfy the diagnostic criteria for AD dementia and DLB, or those for MCI-AD and MCI-LB, they were considered to have mixed disease of AD/DLB. Healthy control (HC) were recruited from an on-going study to recruit normal elderly who does not have any subjective symptoms of cognitive impairment and history of neurological or psychiatric illnesses (Baik et al., 2023). They had normal cognitive function on detailed neuropsychological test, described below, normal neurologic examination, normal brain MRI, and normal findings on ^18^F-Florbetaben PET (FBB-PET), FDG-PET, and DAT-PET scans.

#### 2.1.2 Neuropsychological evaluation

Briefly, this battery included the backward digit span test for attention; Korean version of the Boston Naming Test (K-BNT) for language function; the delayed recall item of Seoul Verbal Learning Test (SVLT) and Rey-Osterrieth Complex Figure Test (RCFT) for verbal- and visual-memory function; and RCFT copy for visuospatial function. Frontal/executive function was evaluated using three groups of tests including motor executive function (contrasting program, go/no-go, fist-edge-palm, alternating hand movement, alternative square and triangle, and Luria loop), phonemic and semantic Controlled Oral Word Association Test (COWAT), and Stroop color reading test. Frontal/executive dysfunction was operationally defined as impairment in at least two of these three groups of tests. Age- and education-matched norms were available for all scorable tests of SNSB, and scores in each specific cognitive test were considered abnormal when they were lower than the −1.0-standard deviation of the norm. Frontal/executive dysfunction was defined as impairment in at least two of the three groups of the tests.

### 2.2 Data acquisition

#### 2.2.1 MRI acquisition

Magnetic resonance imaging (MRI) scans were acquired using a Philips 3.0 T MRI scanner (Philips Achieva; Philips Medical System, Best, The Netherlands) equipped with a SENSE head coil (SENSE factor = 2). T1w images were obtained using a three-dimensional turbo-field sequence with the following parameter: axial acquisition matrix of 224 × 224, reconstructed to 256 × 256 with 170 slices; voxel dimensions of 0.859 × 0.859 × 1 mm^3^; a field of view of 220 mm; echo time of 4.6 ms; repetition time of 9.8 ms; and a flip angle of 8°.

#### 2.2.2 PET acquisition

FDG- and FBB-PET scans were conducted using the Discovery 600 system (General Electric Healthcare, Milwaukee, WI, USA). For FDG-PET, subjects were received an intravenous injection of FDG at a dose of approximately 4.1 MBq per kilogram of body weight. Following a 60-min uptake period, PET images were acquired over a 15-min duration. For FBB-PET, 300 MBq (8 mCi) of FBB was administered intravenously, and imaging was conducted 90 min post-injection over a 20-min session. Image reconstruction was performed using the ordered subset expectation maximization algorithm with four iterations and 32 subsets. A Gaussian filter with a full width at half maximum (FWHM) of 4 mm was applied to reconstructed PET images, producing a 256 × 256 matrix with a pixel size of 0.98 mm and a slice thickness of 0.98 mm.

### 2.3 Image processing

T1w MR and FDG-PET imaging data were pre-processed following protocols detailed below. T1w MR images were bias-corrected, registered to Montreal Neurological Institute (MNI) space, and segmented into tissue types to obtain gray matter (GM) masks. The striatal region masks were integrated into GM probability maps, and a study-specific GM mask was constructed by averaging the GM probability maps across all subjects, followed by excluding voxels with intensity values below 30% of the maximum intensity within the whole brain. FDG-PET images were aligned to the corresponding T1w MR images for each subject and normalized to a cerebellar GM reference region to obtain Standardized uptake value ratio (SUVR) images. Subject Residual Profiles (SRP) images were obtained by log-transforming and double-centering SUVR values within the GM mask (Lee et al., 2022; Moeller and Strother, 1991).

#### 2.3.1 MR image processing

T1w MR images were pre-processed to correct intensity inhomogeneities using the Advanced Normalization Tools (ANTs) N4BiasFieldCorrection algorithm (Tustison et al., 2010). Subsequently, these images were non-linearly registered to the Montreal Neurological Institute (MNI) standard space (average 152 template). Brain region masks were derived for each subject using the HD-BET model (Isensee et al., 2019), and brain tissues were segmented into gray matter (GM), white matter (WM), and cerebrospinal fluid (CSF) using FMRIB’s Automated Segmentation Tool (FAST) (Zhang et al., 2001), which employs a hidden Markov random field model coupled with an Expectation–Maximization algorithm. The striatal regions, including the putamen and caudate, were delineated using the FMRIB Integrated Registration and Segmentation Tool (FIRST) algorithm (Patenaude et al., 2011). These striatal masks were integrated into the GM probability maps for each subject. To construct a study-specific GM mask, the individual integrated GM probability maps were averaged, and all voxels with intensity values below 30% of the maximum intensity within the whole brain were excluded.

#### 2.3.2 PET image processing

FDG-PET images were first linearly registered to the corresponding T1w MR images for each subject using ANTs (Avants et al., 2008). Non-linear transformations computed during T1w image registration were subsequently applied to align the FDG-PET images to the MNI template. Standardized uptake value ratios (SUVRs) were calculated by normalizing the FDG-PET images to the cerebellar gray matter reference region. To isolate the brain region, SUVR images were masked using brain masks generated by HD-BET. No smoothing was applied to the FDG-PET images, as smoothing was found to degrade classification performance. To compute FDG Subject Residual Profiles (FDG-SRP), SUVR values were log-transformed and doubly centered by subtracting the whole-brain SUVR mean value for each subject and the mean value calculated for each voxel profile within the GM mask (Lee et al., 2022; Moeller and Strother, 1991).

### 2.4 Strategies for addressing the data insufficiency problem

Our FDG-PET dataset consists of an insufficient number of samples to effectively train a deep learning model. To address this limitation, we employed three strategies: transfer learning with a pre-trained model called AmyloidPETNet (Fan et al., 2024), expanding the training set by incorporating additional PET datasets (Petersen et al., 2010), and applying data augmentation to the training set (Cubuk et al., 2019).

#### 2.4.1 Transfer learning strategy

The first strategy involves employing pre-training model, a form of transfer learning. Transfer learning is a widely adopted technique in deep learning when training data is insufficient, and studies have demonstrated its effectiveness in improving model performance. For instance, the VGG model, pre-trained on natural image data from ImageNet (Simonyan and Zisserman, 2014), has been successfully applied to AD classification using MRI (Jain et al., 2019; Khan et al., 2019). In such cases, transfer learning facilitates the extraction of useful features by leveraging data that is not directly related to the main task (i.e., AD classification task), ultimately enhancing the model’s performance. Inspired by this approach, we employed AmyloidPETNet, a model pre-trained on amyloid-PET based classification of amyloid positivity to extract meaningful features for our classification task.

#### 2.4.2 AmyloidPETNet

AmyloidPETNet was specifically developed to predict the probability of PET volumes being Aβ positive using 8,476 brain PET scans from 6,722 patients across five different datasets and/or tracers. PET images employed in AmyloidPETNet included five types of amyloid PET tracers: fluorine 18 (^18^F) florbetapir (FBP), carbon 11 (^11^C) Pittsburgh compound B (PiB), ^18^F-florbetaben (FBB), ^18^F-flutemetamol (FMT) and ^18^F-flutafuranol (NAV4694).

#### 2.4.3 Data expansion strategy (ADNI & previous data)

The second strategy involved expanding the training dataset by incorporating additional PET data. The Alzheimer’s Disease Neuroimaging Initiative (ADNI) datasets includes FDG-PET scans labeled for dementia groups such as AD and MCI, as well as non-dementia group like cognitively normal (CN). Although the diagnostic criteria for CN in ADNI may differ from the HC criteria in our dataset, incorporating CN samples from ADNI into our HC samples is expected to facilitate the extraction of meaningful features in our FDG-PET dataset from a transfer learning perspective. For this purpose, we selected 100 CN samples from all ADNI datasets, including ADNI-1, ADNI-2, ADNI-3, and ADNI-GO. To ensure that the age and sex distributions of the extracted ADNI datasets were similar to those of the HC group in our FDG-PET dataset, we divided the age range of the ADNI datasets into five bins, each spanning 6.5 years. We then randomly selected the required number of samples from each bin while considering the sex ratio of the FDG-PET dataset. Similarly, we incorporated out additional PET data labeled for AD, DLB and mixed disease from previous study into the training set. Notably, these additional data were not included in the validation or test sets.

#### 2.4.4 Data augmentation strategy

The third strategy involved applying data augmentation to the training set. We employed three data augmentation techniques: mix-up, flip, and rotation (Cubuk et al., 2019). The mix-up technique involves selecting a random subject from the training dataset that belongs to the same class as the current subject being used for training. The images of the two subjects are then combined using a weighted sum. The coefficients for the weighted sum were determined by a randomly selected alpha value and its complement (1-alpha) from a uniform distribution. For the flip augmentation, we applied a horizontal flip to the images. The rotation augmentation was performed with random angles within a range of up to ±10 degrees. This augmentation method was applied to all training data with a 10% probability during the training phase.

### 2.5 Training the classification model

It is well known that deep learning models require many parameters to extract meaningful features, and training such models requires large datasets. Despite applying various methods to address data insufficiency as described above, the available data remained insufficient to train a large deep learning model. To address this limitation, we developed a classification model using small deep learning networks, alleviating the data insufficiency by training multiple models with fewer parameters instead of a single large model with numerous parameters. In this section, we detail the training methodology for each model and the ensemble approach employed to combine them.

#### 2.5.1 Binary classification model

**Figure 1:**
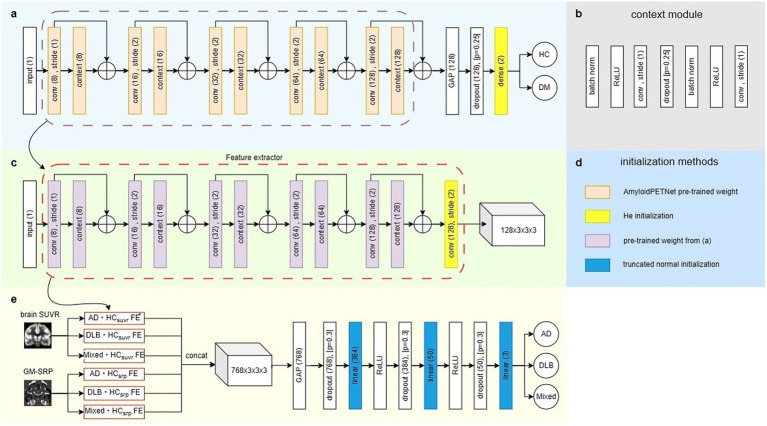
Model architecture in training phase. **(a)** Backbone network structure of the binary classification model modified from AmyloidPETNet. This structure was used for Models 1 through 6 and $\mathrm{AD}\cdot {\text{Mixed}}_{\text{suvr}}$ model. DM represents one of the dementia groups. **(b)** Context module structure used in panel **a**. **(c)** Feature extractor composed of the pre-trained binary classification model with an additional 3D convolution layer. **(d)** Initialization methods applied to the networks in all models. **(e)** $\mathrm{AD}\cdot \mathrm{DLB}\cdot {\text{Mixed}}_{\text{suvr},\mathrm{srp}}$ model architecture, consisting of six feature extractors with an additional linear layer. ^*^FE represents feature extractor.

Based on the finding of previous research (Lee et al., 2022), we anticipated that a simple binary classification model could effectively extract meaningful features for multi-class classification. We trained six deep learning models for binary classification to extract relevant features between the HC group and disease groups. For convenience, we referred to the mixed disease with AD and DLB as Mixed. Specifically, three models were trained using brain-SUVR images to classify AD versus HC, DLB versus HC, and Mixed versus HC, referred to as $\mathrm{AD}\cdot HC_{\text{suvr}}$ , $\mathrm{DLB}\cdot HC_{\text{suvr}}$ , and $\text{Mixed}\cdot HC_{\text{suvr}}$ respectively. Similarly, three additional binary classification models were trained using GM-SRP images, named $\mathrm{AD}\cdot HC_{\mathrm{srp}}$ , $\mathrm{DLB}\cdot HC_{\mathrm{srp}}$ , and $\text{Mixed}\cdot HC_{\mathrm{srp}}$ respectively. As explained in the discussion section, we recognized the necessity of developing an additional binary classification model to classify between AD and Mixed groups. This model, referred to as $\mathrm{AD}\cdot \text{Mixe}d_{\text{suvr}}$ , was trained using the same approach as the other binary classification models and was utilized in the evaluation phase. For model training, we employed AmyloidPETNet as the backbone network. The architectures of these models are shown in Figure 1a, and the context module, which is a key component if the model, is shown in Figure 1b.

We utilized AmyloidPETNet as the backbone network and modified the structure of the final linear layer to 128 × 2 (see Figure 1a). The six AmyloidPETNet models were initialized with pre-trained weights provided by the GitHub repository^1^ for all layers except the final layer, which was initialized using He initialization. The initialization methods for all networks are presented in Figure 1d. Using this backbone model, we trained six binary classification models (i.e., $\mathrm{AD}\cdot HC_{\text{suvr}}$ , $\mathrm{DLB}\cdot HC_{\text{suvr}}$ , $\text{Mixed}\cdot HC_{\text{suvr}}$ , $\mathrm{AD}\cdot HC_{\mathrm{srp}}$ , $\mathrm{DLB}\cdot HC_{\mathrm{srp}}$ , and $\text{Mixed}\cdot HC_{\mathrm{srp}}$ ). For the training and performance evaluation of each model, we utilized only the groups relevant to the specific binary classification task from the entire training set, validation set, and test set. For example, when training the HC versus AD model, only subjects from the HC and AD groups in the training and validation sets were used. Similarly, for calculating the test performance shown in Table 1, only subjects from the HC and AD groups in the test set were included.

**Table 1:** Demographic and clinical characteristics of study participants.

Clinical characteristics	AD	DLB	Mixed	HC	P^1^	P^2^
Number	94	84	72	27	NA	NA
Age, years (SD)	74.0 (7.7)	74.1 (6.6)	73.8 (7.5)	66.3 (8.2)	0.973	<0.001
Female, *N* (%)	54 (57.4%)	49 (58.3%)	54 (75.0%)	15 (55.6%)	0.040	0.073
Education, years (SD)	11.4 (4.7)	9.6 (5.2)	8.9 (5.2)	13.9 (3.6)	0.004	<0.001
*APOE* ε4 carrier^*^, *N* (%)	63 (67.0%)	19 (22.6%)	38 (52.8%)	1 (3.7%)	<0.001	<0.001
Cognitive fluctuations, *N* (%)	0	68 (81.0%)	60 (83.3%)	0	<0.001	<0.001
Visual hallucinations, *N* (%)	0	13 (15.5%)	14 (19.4%)	0	<0.001	<0.001
RBD, *N* (%)	10 (10.6%)	22 (26.2%)	17 (23.6%)	0	0.020	0.001
UPDRS part III (SD)	17.8 (10.8)	19.6 (10.3)	21.1 (11.0)	0.7 (2.1)	0.153	<0.001
K-MMSE (SD)	22.5 (4.1)	22.7 (4.6)	20.2 (5.1)	29.1 (1.3)	NA	<0.001
CDR (SD)	0.6 (0.2)	0.7 (0.4)	0.8 (0.5)	0	<0.001	<0.001
CDR-SOB (SD)	2.7 (2.0)	3.1 (2.7)	4.2 (2.9)	0	<0.001	<0.001
Digit span backward (SD)	74.0 (7.7)	74.1 (6.6)	73.8 (7.5)	66.3 (8.2)	0.035	<0.001
KBNT (SD)	40.1 (10.7)	37.0 (11.2)	35.7 (11.6)	53.3 (4.8)	0.035	<0.001
Rey copy (SD)	28.1 (7.7)	25.6 (8.7)	23.8 (10.4)	34.6 (1.3)	0.008	<0.001
SVLT immediate recall (SD)	13.1 (4.1)	13.6 (4.3)	13.9 (4.7)	27.1 (5.0)	0.572	<0.001
SVLT delayed recall (SD)	1.3 (1.7)	2.1 (2.2)	1.3 (2.0)	9.6 (2.1)	0.005	<0.001
SVLT recognition (SD)	17.0 (2.6)	17.8 (2.8)	16.6 (2.6)	23.0 (1.3)	0.027	<0.001
RCFT immediate recall (SD)	5.2 (4.6)	6.4 (5.6)	3.9 (4.6)	19.9 (6.9)	0.012	<0.001
RCFT delayed recall (SD)	4.3 (4.7)	6.0 (5.9)	3.3 (4.4)	19.7 (6.7)	0.004	<0.001
RCFT recognition (SD)	16.7 (3.0)	17.7 (2.7)	16.2 (2.5)	20.7 (1.9)	0.003	<0.001
COWAT animal (SD)	11.6 (4.4)	10.8 (4.4)	10.1 (3.6)	19.5 (5.3)	0.079	<0.001
COWAT supermarket (SD)	11.1 (4.4)	10.3 (5.4)	9.7 (4.7)	19.9 (5.2)	0.196	<0.001
COWAT phonemic (SD)	19.5 (9.6)	15.0 (8.4)	16.1 (10.9)	38.5 (10.7)	0.007	<0.001
Stroop color reading (SD)	58.1 (31.2)	50.2 (29.2)	47.0 (28.4)	105.9 (12.2)	0.058	<0.001
Abnormal CP, *N* (%)	27 (28.7%)	28 (33.3%)	30 (41.7%)	NA	0.163	NA
Abnormal Go-no-go, *N* (%)	48 (51.1%)	41 (48.8%)	40 (55.6%)	NA	0.549	NA
Abnormal AHM, *N* (%)	45 (47.9%)	42 (50.0%)	43 (59.7%)	NA	0.123	NA
Abnormal AST, *N* (%)	16 (17.0%)	23 (27.4%)	22 (30.6%)	NA	0.083	NA
Abnormal Luria loop, *N* (%)	14 (14.9%)	19 (22.6%)	17 (23.6%)	NA	0.268	NA
Abnormal FEP, *N* (%)	43 (45.7%)	43 (51.2%)	44 (61.1%)	NA	0.089	NA

Data are results of analyses of variance or chi-square tests as appropriate. ^*^APOE genotyping was performed in 93 patients with AD, 84 with DLB, 72 with mixed dementia, and eight HC subjects. SD, Standard deviation; K-MMSE, Korean version of Mini-Mental State Examination; CDR, Clinical Dementia Rating; CDR-SOB, Clinical Dementia Rating Sum of Boxes; K-BNT, Korean version of the Boston Naming Test; UPDRS, Unified Parkinson’s Disease Rating Scale; APOE, apolipoprotein E; RBD, rapid eye movement sleep behavior disorder.

#### 2.5.2 Multi-class dementia classification model

A classification model for AD, DLB, and Mixed was trained using the previously described binary classification models as feature extractors. As shown in Figure 1e, features extracted from the six binary classification models were concatenated and passed through linear layers to predict three dementia classes. We referred to this architecture as $\mathrm{AD}\cdot \mathrm{DLB}\cdot \text{Mixe}d_{\text{suvr},\mathrm{srp}}$ model.

To train dementia classification models, only the AD, DLB, and Mixed groups in training set were used to train a disease classifier. We employed the pre-trained binary classification models, described above, up to the global average pooling layer, composed of five sequential 3D convolution layers and a context module, to extract 128-channel features with a size of 6 × 7 × 6. As shown in Figure 1c, this feature was passed through an additional 3D convolution layer (kernel size = (3 × 3 × 3), stride = 2), resulting in 128 × 3 × 3 × 3 feature. We referred to these architectures as feature extractor from six binary classification models. As shown in Figure 1e, the extracted features from six feature extractor were concatenated along the channel axis, forming a final feature of size 768 × 3 × 3 × 3. This feature was then reduced to a 768 × 1 vector using global average pooling. Subsequently, three linear layers and dropout operations were applied sequentially to reduce the dimensions to 384, 50, and 3, where 3 represents the number of target classes AD, DLB and Mixed. All models in this study were trained independently using cross-entropy loss, and the training and evaluation was conducted using an Nvidia RTX A6000 GPU (48 GB of memory).

### 2.6 Evaluation

**Figure 2:**
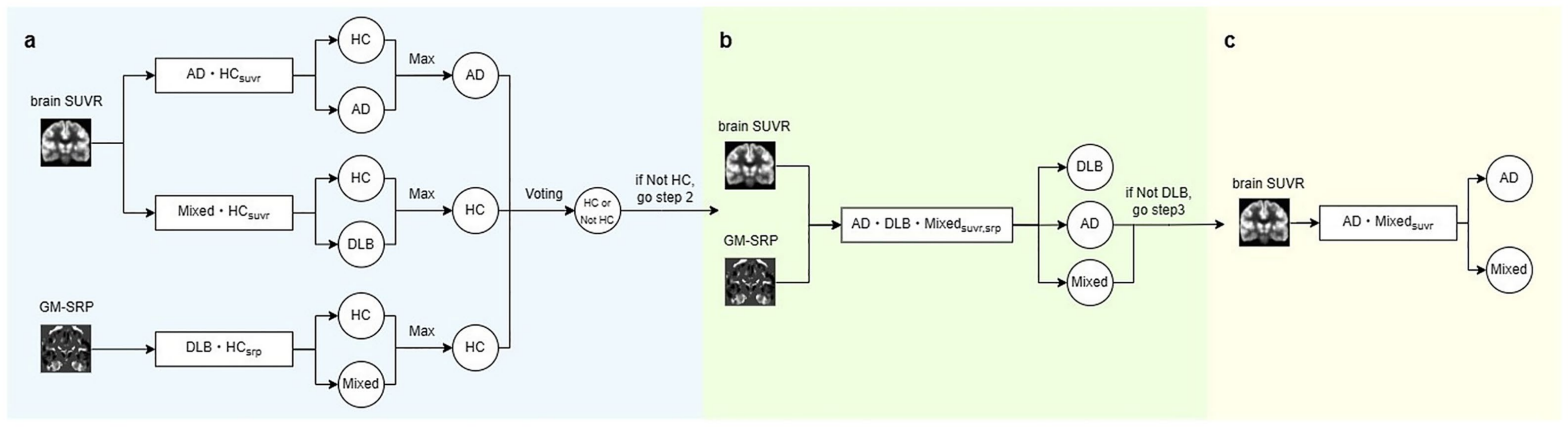
Evaluation steps of the ensemble model. **(a)** Model 1, Model 3, and Model 5 are used to classify the input subject as either HC or a dementia group. The voting mechanism determines the classification as HC only if all three models predict HC; otherwise, if at least one model predicts the subject as belonging to a dementia group, the subject is classified as dementia, and the process proceeds to Step 2. **(b)** For subjects classified as dementia in Step 1, $\mathrm{AD}\cdot \mathrm{DLB}\cdot {\text{Mixed}}_{\text{suvr},\mathrm{srp}}$ model is used to further classify whether the subject belongs to the DLB group or not. The prediction is performed by taking the maximum value from output of $\mathrm{AD}\cdot \mathrm{DLB}\cdot {\text{Mixed}}_{\text{suvr},\mathrm{srp}}$ model. If the subject is classified as AD or mixed instead of DLB, the process proceeds to Step 3. **(c)** For subjects classified as AD or mixed in Step 2, the final prediction between AD and mixed is determined.

The diagnostic performance was evaluated by predicting each subject in the test data using an ensemble of the previously trained models. As shown in Figure 2, the prediction process of the ensemble model consists of three steps. Step 1: The calculated brain SUVR image was passed into $\mathrm{AD}\cdot HC_{\text{suvr}}$ model and $\text{Mixed}\cdot HC_{\text{suvr}}$ model to perform predictions for AD versus HC and Mixed versus HC respectively, while the GM-SRP image was passed into $\mathrm{DLB}\cdot HC_{\mathrm{srp}}$ model to perform predictions for DLB versus HC. If all three models predicted the data as HC, the data was classified as HC, and the prediction process was terminated. Step 2: If one or more models predicted the data as belonging to a dementia group rather than HC, the data was passed to $\mathrm{AD}\cdot \mathrm{DLB}\cdot \text{Mixe}d_{\text{suvr},\mathrm{srp}}$ model for further classification into one of three groups: AD, DLB, or Mixed. If $\mathrm{AD}\cdot \mathrm{DLB}\cdot \text{Mixe}d_{\text{suvr},\mathrm{srp}}$ model predicted the data as DLB, it was classified as DLB, and the prediction process was terminated. Step 3: If $\mathrm{AD}\cdot \mathrm{DLB}\cdot \text{Mixe}d_{\text{suvr},\mathrm{srp}}$ model predicted the data as either AD or Mixed, the data was passed to $\mathrm{AD}\cdot \text{Mixe}d_{\text{suvr}}$ model, which specializes in differentiating between AD and Mixed groups. The prediction from $\mathrm{AD}\cdot \text{Mixe}d_{\text{suvr}}$ model was then used as the final classification result, and the inference process was terminated.

#### 2.6.1 Performance assessment across diagnostic groups categorized by CSF biomarkers

Our performance evaluation is subject to the limitation of a relatively small test dataset. To address this limitation, we further validated the model’s performance using FDG-PET data from the ADNI cohort, where groups were categorized based on CSF biomarker profiles. From the ADNI dataset, we identified subjects who underwent the seed amplification assay (SAA) performed by Amprion Clinical Laboratory (Arnold et al., 2022). To ensure temporal consistency, we included participants whose Elecsys CSF immunoassay (Aβ_42_ and p-tau_181_) and FDG-PET imaging were conducted within 1 year of the SAA (Blennow et al., 2019). Subjects with missing FDG-PET examination dates were excluded to maintain data integrity, resulting in a sample of 618 individuals. Following the recommendations of the ADNI Biomarker Core Steering Committee, AD positivity (AD+) was defined by a CSF p-tau_181_/Aβ_42_ ratio >0.021. DLB positivity (DLB+) was determined by the alpha-synuclein SAA results, specifically utilizing the ‘Detected-1’ label associated with Parkinson’s disease or Lewy body dementia. Participants were categorized into four groups: AD (AD+ and DLB−), DLB (DLB+ and AD−), Mixed (AD+ and DLB+), and HC (AD− and DLB−). The final distribution consisted of 246 AD, 37 DLB, 106 Mixed, and 229 HC subjects. Detailed classification performance metrics are provided in Supplementary Table 2.

## 3 Results

### 3.1 Demographic and clinical characteristics

The HC group was younger, had a longer duration of education, and outperformed the AD, DLB, and mixed disease groups on all cognitive tests (Table 1). Among the AD, DLB, and mixed disease groups, mean age and Unified Parkinson’s Disease Rating Scale (UPDRS) part III scores were comparable. The AD group had a longer duration of education than the DLB and mixed disease group. The proportion of female patients was higher in the mixed disease group compared to the AD and DLB groups, while the proportion of apolipoprotein E (APOE) ε4 carriers was higher in the AD and mixed disease groups than in the DLB group. Cognitive fluctuations and visual hallucinations were observed in the DLB group and mixed disease group, but not in the AD group. The DLB group had a higher proportion of patients with rapid eye movement sleep behavior disorder (RBD) than the AD group. The mixed disease group showed worse performance on the Korean version of Mini-Mental State Examination (K-MMSE), Clinical Dementia Rating (CDR), and CDR- Sum of Boxes (CDR-SOB) compared to the AD and DLB groups. The DLB and mixed disease groups scored lower than the AD group on the digit span backward and semantic Controlled Oral Word Association Test (COWAT) phonemic tasks. In memory-related measures, the AD and mixed disease groups scored lower in Seoul Verbal Learning Test (SVLT) delayed recall, Rey-Osterrieth Complex Figure Test (RCFT) delayed recall, and RCFT recognition compared to the DLB group. Meanwhile, the mixed disease group had lower scores than the AD group in RCFT copy and performed worse than the DLB group in SVLT recognition and RCFT immediate recall.

### 3.2 Classification model performance

**Figure 3:**
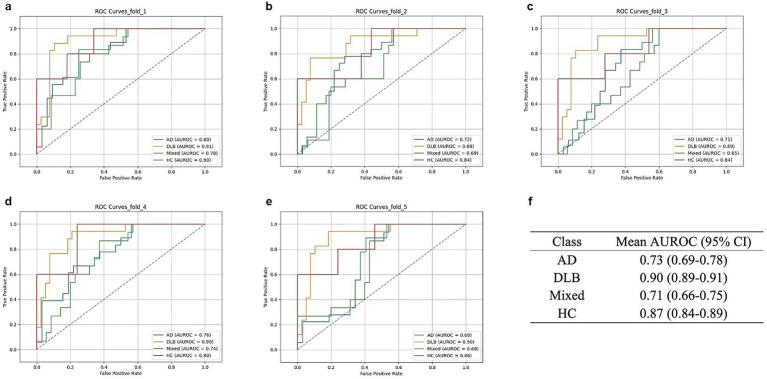
Receiver operating characteristic (ROC) curves across cross-validation. **(a–e)** ROC curves were obtained from the independent test set using models trained on 5-fold cross-validation. Each panel displays fold specific ROC curves for all diagnostic classes: AD (blue), DLB (orange), mixed (green), and HC (red). **(f)** Mean area under ROC (AUROC) across the 5-fold for each class with 95% CI.

**Figure 4:**
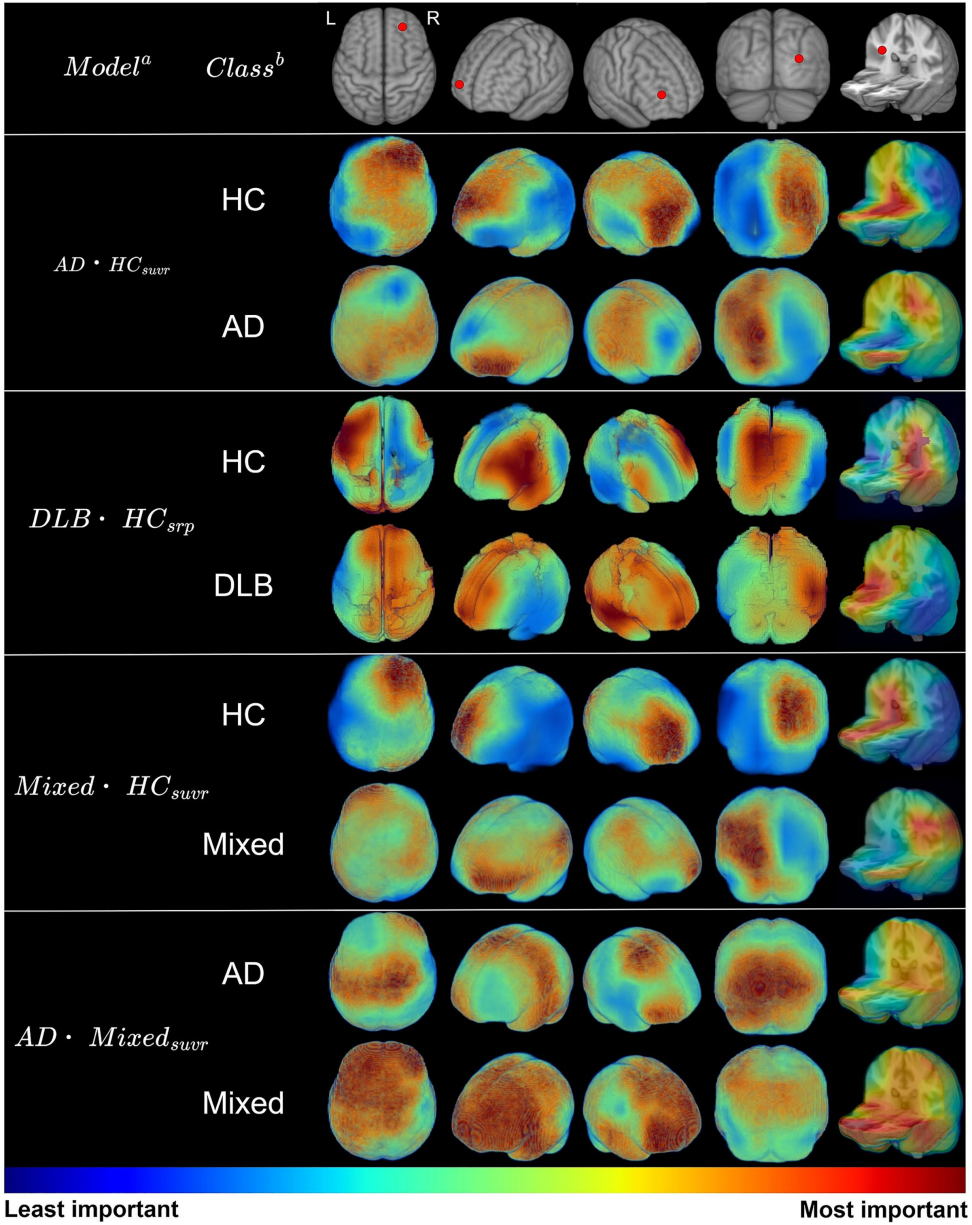
**(a)** Model-level saliency maps are represented only for models included in the evaluation. For each model, we computed subject-level saliency maps for fold-1 test set subjects that were correctly classified and then averaged these maps within each class to obtain class-specific model-level saliency maps. **(b)** The class name for each saliency map. Saliency maps were rendered in neurological orientation and displayed from multiple views. In the top row for each view, the right hemisphere is indicated by a red dot.

In this study, we implemented various classification models and compared the classification performance of them (e.g., $\mathrm{AD}\cdot HC_{\text{suvr}}$ model) (Supplementary Table 1). Although $\mathrm{AD}\cdot \mathrm{DLB}\cdot \text{Mixe}d_{\text{suvr},\mathrm{srp}}$ model is multi-class classification model that predicts one of AD, DLB, or Mixed, its role within the overall ensemble model is primarily to perform binary classification between DLB and Not-DLB (AD or Mixed). Since the classification between AD and Mixed relies entirely on the output of $\mathrm{AD}\cdot \text{Mixe}d_{\text{suvr}}$ , the performance of $\mathrm{AD}\cdot \mathrm{DLB}\cdot \text{Mixe}d_{\text{suvr},\mathrm{srp}}$ model reflects its binary classification performance between Not-DLB (AD or Mixed) and DLB (Supplementary Table 1). The ROC curves of the ensemble model are shown in Figure 3, and the detailed class-specific performance metrics are shown in Table 2. To evaluate the generalization performance of our model, we additionally conducted a 5-fold cross-validation. The saliency maps for all models included in the ensemble model, except for the $\mathrm{AD}\cdot \mathrm{DLB}\cdot \text{Mixe}d_{\text{suvr},\mathrm{srp}}$ model, are shown in Figure 4, highlighting the brain regions most influential for disease prediction. For each model, subject-level saliency maps were first computed for correctly classified subjects in the fold-1 test set. These subject-level saliency maps were then averaged within each class to obtain class-specific model-level saliency maps, resulting in two model-level saliency maps for each binary classification model. All saliency maps were generated using the Gradient-weighted Class Activation Mapping (Grad-CAM) (Selvaraju et al., 2017), then normalized to a range between 0 and 1. From these normalized saliency maps, regions of interest (ROIs) with values exceeding 0.7 were identified and are presented in Table 3, with all ROI definitions following the AAL3 template (Rolls et al., 2020). Saliency map and ROI for the $\mathrm{AD}\cdot \mathrm{DLB}\cdot \text{Mixe}d_{\text{suvr},\mathrm{srp}}$ model are not reported, as this model aggregates features from multiple feature extractors, making its interpretation less meaningful.

**Table 2:** Ensemble model classification performance.

Method	Class	Sensitivity	Specificity	PPV	NPV
van Gils et al. (2024)	AD	**0.70**	**0.92**	**0.87**	0.79
	DLB	0.42	0.90	0.17	**0.96**
	DLB/AD+^a^	0.29	**0.89**	0.12	**0.97**
	HC	0.77	0.91	0.88	0.82
Proposed^b^	AD	0.67 (0.58–0.76)	0.78 (0.75–0.81)	0.60 (0.57–0.63)	**0.83** (0.79–0.87)
	DLB	**0.78** (0.75–0.81)	**0.93** (0.92–0.94)	**0.83** (0.81–0.85)	0.91 (0.90–0.92)
	Mixed	**0.48** (0.43–0.53)	0.83 (0.79–0.87)	**0.52** (0.46–0.58)	0.77 (0.70–0.84)
	HC	**1.00** ^c^	**1.00**	**1.00**	**1.00**

The table shows class-wise classification performance for each model. ^a^This prior study used MRI data and did not considering AD copathology in DLB patients, which differs from our approach. However, to our knowledge, it is the only study that has performed multiclass classification of AD, DLB, Mixed, and HC, so we used it as a reference for performance comparison. ^b^Averaged 5-fold cross-validation results of the proposed method. ^c^CI for HC group was not reported because standard deviation is zero. The better-performing method between the two is highlighted in bold.

**Table 3:** Highlighted brain regions identified in saliency maps across classification models.

Model^a^	ROI^b^
$\mathrm{AD}\cdot {\mathrm{HC}}_{\text{suvr}}$	bilateral: hippocampus, putamen, thalamus, occipital cortex, frontal lobe left: parietal cortex, fusiform, lingual, calcarine
$\mathrm{DLB}\cdot {\mathrm{HC}}_{\mathrm{srp}}$	bilateral: pallidum, temporal cortex, putamen, insula right: heschl, caudate left: amygdala, occipital sup, rolandic, hippocampus, calcarine, thalamus, lingual, cuneus
$\text{Mixed}\cdot {\mathrm{HC}}_{\text{suvr}}$	bilateral: occipital cortex, calcarine, frontal lobe right: pallidum, hippocampus, lingual, precuneus left: parietal cortex
$AD\cdot \mathrm{Mix}ed_{\text{suvr}}$	bilateral: frontal lobe, putamen, caudate, amygdala, anterior cingulate cortex, occipital lobe, calcarine, lingual, motor cortex right: temporal lobe

Summary of regions of interest (ROIs) identified from Grad-CAM saliency maps generated by classification models. ^a^For each model, we computed model-level saliency maps that were provided in Figure 4. While various classification models were investigated, only those included in the evaluation are represented, with the expectation of the $\mathrm{AD}\cdot \mathrm{DLB}\cdot \text{Mixe}d_{\text{suvr},\mathrm{srp}}$ model. ^b^All ROIs listed in the table correspond to regions with saliency values normalized within the range from zero to one; only ROIs with saliency values greater than 0.7 are reported. ROI definitions follow the AAL3 template (Rolls et al., 2020). Regions identified in both hemispheres are reported as bilateral, whereas those identified only in a single hemisphere are reported as right or left.

## 4 Discussion

We developed the classification model based on findings from several prior studies related to DLB. This section outlines the results and interpretations of these studies, as well as the strategy applied in developing our model. Previous research demonstrated that AD, DLB, and Mixed groups commonly exhibit hypermetabolism in brain regions such as motor cortex, pallidum and cerebellum while showing hypometabolism in the parietal and inferior/lateral temporal cortices, compared with the HC group (Lee et al., 2022). Our six binary classification models were designed to leverage these characteristics. Specifically, we hypothesized that the distinct features of the AD, DLB, and Mixed groups would be well captured by training deep learning models to classify HC versus dementia groups, as these features are prominently observed when compared to the HC group.

However, as mentioned earlier, a significant challenge is that deep learning models require large amounts of data to be trained effectively to extract appropriate features. To address the issue of limited data availability, we applied transfer learning using the AmyloidPETNet, a model pre-trained on a large-scale Amyloid PET dataset. Although the pre-trained weights of AmyloidPETNet were originally trained using amyloid PET data, which exhibit distinct characteristics compared to our FDG-PET data, the pre-trained weights provided a robust initialization that facilitated the model training on the limited dataset, mitigating potential training challenges and ensuring stable model convergence.

According to prior research, notable differences in metabolic patterns are observed between disease groups and the HC group across multiple brain regions, suggesting that classification between HC and disease groups may be relatively straightforward (Lee et al., 2022). In contrast, differentiating among AD, DLB, and Mixed groups remains more challenging. For instance, metabolic patterns in regions such as the cerebellum, pallidum, motor cortex, inferior/lateral temporal cortex, and parietal cortex effectively differentiate AD from HC, yet these regions offer limited discriminative power for separating AD, DLB, and Mixed groups. Because the regions that exhibit discriminative metabolic patterns for classifying AD, DLB, and Mixed groups are limited, training a model to distinguish among the three groups simultaneously is challenging.

To address this issue, we developed a classification model specifically designed to distinguish the DLB group from the AD and Mixed groups, based on findings from previous studies. According to prior research, metabolic patterns in regions such as the hippocampus, entorhinal cortex, and medial occipital cortex may offer meaningful discriminative power for separating the DLB group from the AD and Mixed groups (Lee et al., 2022). Consequently $\mathrm{AD}\cdot \mathrm{DLB}\cdot \text{Mixe}d_{\text{suvr},\mathrm{srp}}$ model was expected to effectively distinguish the DLB group from the AD and Mixed groups. Subsequently, we trained an additional binary classifier, referred to $\mathrm{AD}\cdot \text{Mixe}d_{\text{suvr}}$ , specifically designed to classify the AD and Mixed groups.

As described above, our evaluation process consisted of three steps. The purpose of Step 1 is to classify HC and dementia, and for this purpose, we utilized three of the six trained binary classification models capable of performing this task. This strategy was based on previous research that AD and Mixed group characteristics are better represented in SUVR images, while DLB characteristics are better represented in SRP images (Kang et al., 2024). The binary classification models $\mathrm{AD}\cdot HC_{\mathrm{srp}},\mathrm{DLB}\cdot HC_{\text{suvr}}\;\text{and Mixed}\cdot HC_{\mathrm{srp}},$ which were not included in the evaluation, were used solely as feature extractors during training of the $\mathrm{AD}\cdot \mathrm{DLB}\cdot \text{Mixe}d_{\text{suvr},\mathrm{srp}}$ model (Figure 1e).

To the best of our knowledge, no prior study has performed multiclass classification of AD, DLB, Mixed and HC using brain imaging data although one study using brain MRI performed a similar analysis (van Gils et al., 2024). In that study, the Mixed group definition accounted for AD co-pathology in DLB cases (DLB/AD+) but not for DLB co-pathology in AD cases, making direct comparison with our results difficult. Nevertheless, we include their classification performance in Table 2 for reference, as their approach is most comparable to ours.

To further validate the model’s performance, we conducted an additional evaluation using the ADNI dataset, where groups were categorized based on CSF biomarkers. Although a direct comparison is challenging due to the inherent discrepancy in classification criteria between our dataset and the ADNI cohort, it serves as an informative indirect assessment. As presented in Supplementary Table 2, an overall decline in diagnostic performance was observed. This performance degradation can be attributed to two primary factors. First, the cohort bias between the FDG-PET images in our clinical collection and those from the ADNI dataset remained uncorrected. Second, FDG-PET imaging does not directly visualize the deposition of α-synuclein, amyloid-β(Aβ), or tau proteins. While our group classification was established by integrating DAT-PET findings with clinical features such as cognitive fluctuations and visual hallucinations, biomarker-based classification focuses strictly on protein pathology, which naturally results in a discrepancy in model performance. Nevertheless, the fact that the model’s performance remained well above chance levels suggests its capacity to capture FDG-PET metabolic features that correlate with protein-related pathologies, despite the absence of explicit training on biomarker-stratified labels.

To explore the brain regions influencing classification decisions, we generated saliency maps using Grad-CAM, a widely used explainable AI (XAI) technique (Figure 4). Table 3 present the brain regions that each model attended to during classification. The identified regions and their corresponding saliency values are not directly interpretable in terms of specific biological meaning, but instead represent the areas to which the models attended when learning to classify groups based on the data. Notably, the models focused on regions such as the striatum (putamen and caudate), amygdala, and occipital cortex, which aligns closely with findings from previous studies. The striatum is well-established as a region strongly correlated with α-synuclein concentration and dopaminergic neurodegeneration in patients with DLB, distinguishing it from AD (McKeith et al., 2017). In the striatum of DLB patients, the loss of dopamine transporters due to α-synuclein concentration is a robust biomarker. Amygdala acts as a primary hub for the accumulation and propagation of various proteins, showing distinct α-synuclein pathology between DLB and AD with amygdala-restricted Lewy bodies (AD/ALB) (Nelson et al., 2018; Sorrentino et al., 2019). Furthermore, the hypometabolism in the occipital lobe is a well-established biomarker for DLB and is closely associated with visual hallucinations (McKeith et al., 2017).

The SRP images in the training dataset were centered twice: first using each individual’s whole-brain mean SUVR value and then using the group-specific voxel-wise mean SUVR values. However, during the test phase, the group of the input data is unknown, making it impossible to apply centering procedure using the voxel-wise mean values of the dementia or control group. As a workaround in the test phase, the voxel-wise mean SUVR values of the entire training dataset were used for centering. This approach, however, may not be entirely appropriate.

Our classification model demonstrates lower sensitivity in the Mixed group compared to other groups. However, it effectively distinguishes the DLB group from the AD and Mixed groups. Previous autopsy research indicates that the Mixed group presents with metabolic deficits that closely mimic AD (Silva-Rodriguez et al., 2023). This diagnostic challenge likely contributed to the relatively low sensitivity observed in the Mixed group, despite our implementation of a specialized sub-model designed to distinguish it from the AD group (Figure 2c). The relatively low performance suggests that the current dataset may be inadequate for the model to extract discriminative high-dimensional information between AD and Mixed cases. Expanding the training cohort through ongoing data acquisition will likely be essential to improve the model’s sensitivity in identifying the Mixed group. Also, the challenges in differentiating between the Mixed and AD groups could potentially be mitigated by increasing the availability of FDG-PET data and incorporating additional modalities such as electroencephalography (EEG).

The classification model developed in this study is the first automated model capable of distinguishing among AD, DLB, Mixed, and HC groups. By facilitating the identification of DLB and Mixed cases, which are frequently underdiagnosed in real-world clinical settings, the model may support more accurate diagnostic decision-making and facilitate the development of appropriate treatment strategies.

## Data Availability

The raw data supporting the conclusions of this article will be made available by the authors without undue reservation.

## Footnotes

1
https://github.com/sotiraslab/AmyloidPETNet

## Group member of Alzheimer’s Disease Neuroimaging Initiative

A complete listing of ADNI investigators can be found at: http://adni.loni.usc.edu/wp-content/uploads/how_to_apply/ADNI_Acknowledgement_List.pdf.
