# Supplementary material for: Deep learning for FDG-PET classification in patients with Alzheimer’s disease, dementia with Lewy bodies and their mixed pathology: a solution for diagnostic heterogeneity
Source: Front Aging Neurosci. 2026 Mar 5;18:1780858. doi: 10.3389/fnagi.2026.1780858 (PMC12999798; doi:10.3389/fnagi.2026.1780858)
Supplement: Supplementary file 1 [file Data_Sheet_1.docx]

Supplementary Material

Supplementary Table 1. Individual classification model performance

| Model^a^ | Accuracy | AUROC | Sensitivity | Specificity | PPV | NPV |
| --- | --- | --- | --- | --- | --- | --- |
| $\mathrm{AD}・HC_{\mathrm{suvr}}$ | 0.90  (0.89-0.92) | 0.91  (0.88-0.93) | 0.98  (0.95-1.00) | 0.64  (0.56-0.72) | 0.91  (0.89-0.93) | 0.91  (0.80-1.00) |
| $\mathrm{DLB}・HC_{\mathrm{suvr}}$ | 0.94  (0.91-0.96) | 0.97  (0.95-0.99) | 0.98  (0.93-1.00) | 0.80  (0.68-0.92) | 0.95  (0.91-0.98) | 0.94  (0.83-1.00) |
| $\mathrm{Mixed}・HC_{\mathrm{suvr}}$ | 0.91  (0.89-0.93) | 0.99  (0.97-1.00) | 0.99  (0.96-1.00) | 0.68  (0.52-0.84) | 0.91  (0.86-0.95) | 0.97  (0.90-1.00) |
| $\mathrm{AD}・HC_{\mathrm{srp}}$ | 0.83  (0.80-0.87) | 0.81  (0.71-0.91) | 0.91  (0.87-0.95) | 0.56  (0.48-0.64) | 0.88  (0.87-0.90) | 0.65  (0.56-0.75) |
| $\mathrm{DLB}・HC_{\mathrm{srp}}$ | 0.84  (0.81-0.86) | 0.74  (0.69-0.79) | 0.89  (0.85-0.94) | 0.64  (0.56-0.72) | 0.90  (0.88-0.91) | 0.65  (0.58-0.73) |
| $\mathrm{Mixed}・HC_{\mathrm{srp}}$ | 0.89  (0.87-0.91) | 0.89  (0.80-0.97) | 0.95  (0.92-0.97) | 0.72  (0.62-0.82) | 0.91  (0.89-0.94) | 0.83  (0.74-0.92) |
| $\mathrm{AD}・\mathrm{DLB}・\mathrm{Mixe}d_{suvr,srp}^{b}$ | 0.87  (0.86-0.88) | 0.91  (0.89-0.92) | 0.79  (0.76-0.82) | 0.92  (0.90-0.93) | 0.83  (0.81-0.85) | 0.89  (0.88-0.91) |
| ${AD・\mathrm{Mix}ed}_{suvr}$ | 0.65  (0.60-0.71) | 0.62  (0.54-0.70) | 0.73  (0.65-0.82) | 0.54  (0.49-0.59) | 0.67  (0.62-0.72) | 0.62  (0.57-0.68) |

Abbreviations: AUROC = Area Under Receiver Operating Characteristic, PPV = positive predictive value, NPV = negative predictive value, TP = number of true positives, TN = number of true negatives, FP = number of false positives, FN = number of false negatives, P = total number of positives, N = total number of negatives.

^a^ All classification models developed in this study. Models trained on each fold were applied to the independent test set and performance metrics were calculated. The metrics are defined as follows: Accuracy = $\frac{\boldsymbol{TP+TN}}{\boldsymbol{P+N}}$, Sensitivity = $\frac{\boldsymbol{TP}}{\boldsymbol{TP+FN}}\boldsymbol{,}$ Specificity =$\frac{\boldsymbol{TN}}{\boldsymbol{TN+FP}}\boldsymbol{,}$PPV = $\frac{\boldsymbol{TP}}{\boldsymbol{TP+FP}}$, NPV = $\frac{\boldsymbol{TN}}{\boldsymbol{TN+FN}}$. Metrics were averaged across the 5-fold with 95% CI. ^b^ The ${\mathbf{AD}\mathbf{・}\mathbf{DLB}\mathbf{・}\mathbf{Mixe}\boldsymbol{d}}_{\boldsymbol{suvr,srp}}$ model, although intrinsically a multi-class classification model, was applied in the final ensemble model to classify DLB versus not-DLB where not-DLB includes AD and Mixed groups, and thus, its metrics were calculated based on this binary classification task.

Supplementary Table 2. Model performance for CSF biomarker-defined groups in the ADNI cohort.

| Method | Class | Sensitivity | Specificity | PPV | NPV |
| --- | --- | --- | --- | --- | --- |
| Proposed | AD | 0.59  (0.55-0.63) | 0.84  (0.82-0.86) | 0.71  (0.67-0.75) | 0.76  (0.73-0.78) |
|  | DLB | 0.62  (0.57-0.67) | 0.94  (0.93-0.95) | 0.39  (0.34-0.44) | 0.97  (0.97-0.98) |
|  | Mixed | 0.34  (0.31-0.38) | 0.85  (0.84-0.86) | 0.32  (0.29-0.35) | 0.86  (0.85-0.87) |
|  | HC | 0.81  (0.78-0.84) | 0.86  (0.85-0.88) | 0.77  (0.75-0.80) | 0.88  (0.87-0.90) |

The table shows the classification performance for the ADNI dataset groups, as defined by CSF biomarker profiles. Each metric represents the average performance evaluated using model weights from a 5-fold cross-validation scheme trained on this study's dataset.
